# Book Review

**Published:** 1922-09

**Authors:** 


### Reviews of Books.

**The Spleen and some of its Diseases.** By Sir BERKELEY MOYNIHAN. Pp. xi., 129. Bristol: John Wright & Sons Ltd. 1921. Price 21s. net.—In this book Sir Berkeley Moynihan has reprinted, with considerable expansions, his Bradshaw Lecture for 1920. As is to be expected, the writer's chief concern is to define the uses and limitations of the surgical treatment of diseases of the spleen. After a brief account of the anatomy of the spleen and the history of splenectomy, he summarises in a readable and highly suggestive chapter much of our present knowledge as to the functions of the spleen. This is followed by a brief chapter on splenic pathology, and an interesting general account of the symptomatology of splenic disease. The remainder of the book deals *seriatim* with the leukæmias, Hodgkin's disease, and other forms of splenic disease. The text is elucidated by some interesting schematic drawings, for which Sir Berkeley Moynihan expresses indebtedness to Dr. Oscar Gruner, his pathological colleague.

**The Early Diagnosis of the Acute Abdomen.** By ZACHARY COPE, B.A., M.D., M.S Lond., F.R.C.S. Pp. xv., 223. London: Oxford Medical Publications. 1921. Price 12s. 6d. net.—As the title implies, this book does not concern itself with details of treatment, as in almost all the conditions referred to early operative interference is regarded as necessary if the best results are to be obtained. It is essentially a book of diagnosis, and an attempt is made to differentiate the symptoms of acute disease referable to the various viscera, for, as the author states, "earlier diagnosis means better prognosis." To this end tabular statements and diagrams are introduced and various signs elaborated. Some of these last are new to us, and others are not as well known as they deserve to be. Boas's sign does not, however, appear to be mentioned. The book is didactic in tone, and for teaching purposes has the merit of clearness and forcefulness of expression. The tyro might believe that when armed with such knowledge mistakes in diagnosis could scarcely occur; but the surgeon of experience will remember, perhaps with sadness, that the aggregation of symptoms designated "peritonism" by Treves, and occurring in most acute abdominal crises is frequently attended by great difficulty in ascertaining the particular organ which—in the early stage—is the cause of the trouble. The author has, however, successfully put before us an honest attempt to help us in our difficulty ; his teaching is sound, his investigation of cases is thorough, and his conclusions are founded on scientific principles.

**Lectures on Surgery of the Stomach and Duodenum.** By JAMES SHERREN, C.B.E., F.R.C.S. Pp. 96. London : H. K. Lewis & Co. Ltd. 1921. Price 4s. 6d. net. These lectures were delivered to students of the London Hospital, and are accordingly didactic in style. The lesson it is intended to convey is often pithily put in a direct manner, as shown in the following sentences : " Never perform gastro-jejunostomy, it is not a cure for acute ulcer." " Plastic operations on the stomach, such as gastro-gastrostomy, should not be done, they have no curative effect." " Never consider fat necrosis as pathognomonic of acute pancreatitis." " You may diagnose appendix dyspepsia ; you should never act on that diagnosis . . . but search elsewhere." The value attached to X-rays in the diagnosis of gastric ulcer on pages 16 and 23 respectively are not so happily worded, and appear contradictory. The work is a concise account of modern knowledge in the subject of which it treats, and is exemplified by cases in the author's experience. The teaching is sound, and may be safely taken as a guide to the class of operation required in such diseases. The actual surgical procedures are not described in detail. There is a brief index.

**A Guide to Urinary Diseases.** By ADOLPH ABRAHAMS, O.B.E., M.D. Cantab., and A. CLIFFORD MORSON, O.B.E., F.R.C.S. Eng. Pp. vii., 120. London : Edward Arnold & Co. 1921. Price 9s. net.—This will be found a useful book for the general practitioner so often called upon to distinguish and identify the medical from the surgical renal lesion. While details of investigation and treatment have been given for urgent cases within his province, the authors have wisely omitted these where the disease calls for special knowledge and experience. The essential pathological details are included, and practically all abnormalities in the urine have been considered. Although some valuable minor points in treatment, within the practitioner's scope, do not appear, the book will be found to repay perusal.

**The Surgery of the Peripheral Nerve Injuries of Warfare.** By HARRY PLATT, M.S., F.R.C.S. Pp. 49. Bristol : John Wright & Sons Ltd. 1921. Price 4s. net.—It is in a measure unfortunate that the publication of this book should have been delayed until a time when the general interest in the nerve injuries of warfare has largely died out, because it is undeniable that they present quite a different problem from nerve injuries in civilian practice, to which interest has with most surgeons returned. The writer speaks from a large experience; he has operated on over 500 nerve cases, and has taken much trouble to keep in touch with the end-results. There is a good description of the physiology and pathology of nerve injuries, which does not include anything very novel. The writer, like most others, is sceptical as to the value of the classical signs of electrical reaction of degeneration, but attaches great importance to direct stimulation of the nerve exposed at operation. He points out that sometimes one finds a response in the muscles supplied by twigs coming off close below the site of injury, when sufficient time has elapsed for some recovery to take place, but that even after a very long wait the distal muscles do not respond. Turning to details of operative treatment, he is very doubtful of the value of mere neurolysis. Like everyone else, he finds all other methods very inferior to direct end-to-end suture, and various devices are described to facilitate this in difficult cases. A table of end-results is given, which shows, taking them all together, 79 per cent. recoveries and 21 per cent. failures. In many cases, however, the degree of recovery is by no means so satisfactory, from the point of view of functional efficiency, as we used to expect. The book is pleasantly written, well got up, and has some good illustrations.

**Aids to Operative Surgery.** By H. C. ORRIN, O.B.E., F.R.C.S. Ed. Pp. viii., 236. London: Baillière, Tindall and Cox. 1921. 6s. net.—This is a new addition to the popular Aid series. It has been written for the student revising for his final examination. The book is divided up regionally into seven sections dealing with the abdomen, the female pelvis, head and neck, arm, chest, leg, and a chapter on the spine, arteries, tendons and nerves. The ear and nose are included, but not the eye. A mass of information has been compressed into a small space. This makes the subject-matter difficult to digest unless the student has previously seen or read about the operation. This the author in his preface assumes to have been done. There are no illustrations, a feature in which most recent works on operative surgery have shown great advance. The steps of each operation are well described. Knowledge acquired from the war has been made use of to the extent of the various methods of blood transfusion and nerve suture, but no mention is made of recent work on pedicled skin flaps. The book is provided with an index, and is of a suitable size to carry in the coat pocket.

**Symptomatology, Psychognosis and Diagnosis of Psychopathic Diseases.** By BORIS SIDIS, M.D. Pp. xix., 448. Edinburgh: E. & S. Livingstone. 1921. Price 21s. net.—

The author writes in an interesting, dogmatic style. In the introduction he opens with a very legitimate plea for a better general acquaintance on the part of the profession with psycho-pathology. He indulges in a bitter diatribe against psycho-analysis, which would be more convincing if it were not so obviously biased. In claiming great originality for his own views the author quite overlooks what he owes to Freudian psychology. There is an excellent account of the hypnoidal twilight state ; but one stops to wonder why it should be called the hypnoidal state of Boris Sidis. The chapters on hallucination and hypnotic hallucination are particularly interesting. The author's account of the aphasias is rather confused. It is really the old physiological theory of Wernicke elaborated on psychological lines. Since this theory can hardly be seriously upheld nowadays, elaboration of it is not profitable. The early part of the chapter on amnesia is excellent, but the author's later remarks show signs of confusion similar to that found in his exposition of aphasia. He extends the use of the term amnesia beyond what is usually accepted. Many will readily agree with the remarks on Freudian symbolism found on page 303. There is little new in Part III. of the book, but taken as a whole it is well worth reading.

**Therapeutic Immunization in Asylum and General Practice.** By W. FORD ROBERTSON, M.D. Pp. vii., 278. Edinburgh : E. & S. Livingstone. 1921. Price 15s. net.—An original and useful book, not adapted or designed to supersede standard manuals of bacteriological technique, but especially valuable in disclosing the impressions made on the author's mind on a multitude of points of the utmost practical importance and suggestiveness, by ten years of laborious work in actual investigation and practice of active immunization on a large scale. " Numerous facts new to science are recorded," he tells us in the preface, " and many heterodox opinions are expressed that are not likely to be accepted without a fight." This is quite as it should be, and very welcome to those of us who are tired of seeing manual after manual appear as like as peas in a pod and disclosing industrious compilation in a much greater degree than individual experience. Faithful and painstaking observation and record of unexpected or novel phenomena which occur in the investigation and treatment of almost every case which is carefully studied are the most fruitful bases of advance in knowledge, even when the conclusions at first drawn from them may be partial or premature. Many of the observations noted in this book await corroboration by other workers, *e.g.* " a fact worth noting is the apparently constant prominence of ærobic or anærobic diphtheroid bacilli in the intestinal tract of persons suffering from psoriasis " (p. 161). The author, as is well known, advocates a toxic theory of insanity, and as regards dementia præcox finds three forms of infection specially prominent: neurotoxic diphtheroid bacilli, pneumococci and neurotoxic anærobic intestinal streptothrices. He goes so far as to say "evidence has been obtained that if the chronic bacterial infections in cases of dementia præcox in the early stage are properly treated by therapeutic immunization, the patient benefits both in bodily and mental health." Yet there is no mention of improvement in five of the six illustrative cases cited; as to the remaining one, we are told "the patient steadily improved and was discharged after six months. He had been resident in the Asylum for three months before treatment was begun without any improvement taking place." But we are not informed as to any concurrent treatment, and the psycho-analysts, with whom our author breaks a lance or two, would certainly like some enlightenment on this point. "Rheumatoid" arthritis Dr. Robertson considers due in about 60 per cent. of the cases to a special type of pneumococcus, and in about 40 per cent. to "anærobic gonococci." His evidence is based on the frequent occurrence of these organisms in the usual foci of infection, the intense focal reactions to inoculation by these vaccines and the degree of improvement following the treatment. With the following remarks the reviewer is in full agreement: "Therapeutic immunization in any case of rheumatoid arthritis, if it is to be satisfactory, must be based upon a very exhaustive bacteriological investigation. Every possible source of infection must be explored, and all pathogenic bacteria occurring as the agents of a chronic infection must be dealt with. As a matter of routine, the possibility of there being a gonococcus infection must be investigated with the utmost care." We may add that tuberculous and syphilitic factors should, as a matter of routine, also receive attention, even when there is nothing in the "clinical picture" to implicate these factors. The descriptions of technique in the book are in some cases rather scanty, yet there are many practical hints of value. A book which will well repay study and the exercise of the critical faculty.

**An Introduction to Dermatology.** By NORMAN WALKER, M.D. Seventh Edition. Pp. xviii., 366. Edinburgh: W. Green & Son Ltd. 1922. Price 21s. net.—With a few additional illustrations and only a little added to the scope of the work, this excellent text-book is republished in its seventh edition. Its clearness of description and its excellent balance makes this the premier text-book for students in dermatology. Dr. Walker has resisted, advisedly in our opinion, an increase in the size of the book. Others have produced large and expensive editions of their works, but this has already reached the utmost limit of the student's text-book. It is to be hoped that the aim of subsequent editions will be to retain the small size and to leave the discussion of unripe theories to library volumes.

**Skin Diseases in General Practice.** By HALDIN DAVIS, M.B. Pp. xii., 355. London: Oxford Medical Publications. 1921. Price 25s. net.—The second edition of Haldin Davis's *Diseases of the Skin in General Practice* has added material in the treatment of syphilis and ringworm. This has added to the value of the work, but it might still be fuller in detail in the matter of the dosage of Neo-Salvarsan used in the injection courses in syphilis or in the intramuscular method of administration. There are other small and relatively unimportant omissions, but the book serves a useful purpose in that the subject-matter has a regional arrangement which makes it easy of reference where diagnosis of a local disease is undecided. It is sound in its teaching, and can be recommended as a good clinical text-book on dermatology.

**The Physiology of Gout, Rheumatism and Arthritis as a guide to accurate diagnosis and efficient treatment.** By PERCY WILDE, M.D. Pp. 229. Bristol: John Wright and Sons Ltd. 1921. Price 12s. 6d.—The author of this book devotes the first half of the volume to a discussion on the accepted views of the part played by uric acid in gout. He is at some pains to explain that he is not a chemist, so that his criticisms of those workers who have not suffered from this disqualification are of little moment. In the latter half of the book we have been deeply interested with the wealth of careful clinical observations and the sound diagnostic skill shown everywhere. There are refreshing digressions too, such as that on the effect of alcohol in gout, where we find observations leading in the direction least to be expected. The author is interesting also on the subject of heating of rooms, and on writers who see bacteria in everything without explaining their action. He has no sympathy with the lax observers who confound acute rheumatic gout and rheumatoid arthritis, and he expends much ingenuity and time in showing how his pathological theories can explain the facts. The book is well printed on good paper, and the price in these days is surprisingly moderate.

**Electro-therapeutics for Practitioners.** By FRANCIS HOWARD HUMPHRIS, M.D. Second Edition, revised and enlarged. Pp. x., 300. London: Oxford Medical Publications. 1921. Price 21s. net.—This is one of the most interesting and suggestive books on the subject which we have met with. It is not a general treatise but a delightful account of the methods which the author in a long experience has found effective, the diseases in which he has met with most success, and not least the lessons learnt from failures. We do not accept all his conclusions, indeed we think that more vigorous methods of proof, logical and statistical, are required in statements upon electrical treatment, than those which he and most writers on the subject deem sufficient. The use of static electricity, and especially of the wave current, is described at length, since the author has found it more efficacious than any other form. Radiant heat, diathermy, and X-ray treatment are also discussed in detail. The chapters on diseases amenable to electrical treatment afford us many original methods and observations, one not the least important being the classification and treatment of abnormal blood pressures. He finds that while the hypertension of nephritis is best treated by radiant heat baths, ordinary hypertension can usually be reduced by high-frequency treatment and kept down to a lower figure for years. One would much like statistics in such an important matter on which thousands of lives depend, while the theoretical and unprovable discussion of how a current may act upon tissues leaves us uninterested. Although we are spared the usual descriptions of common batteries, special modifications, apparatus and methods not described in ordinary text-books are discussed in detail, which adds greatly to the value of the book, making it a mine of information in the less known methods of treatment.

**The Anatomy of the Human Orbit and Accessory Organs of Vision.** By S. Ernest Whitnall, M.D., B.Ch. Pp. xi., 428. London: Henry Frowde and Hodder & Stoughton. [1921.] Price 35s. net.—This book, which is one of the Oxford Medical Publications, is founded on lectures for the diploma of ophthalmology. It consists of 4 parts, (1) The bones, (2) The soft parts outside the eyeball, (3) The eyeball, (4) The cerebral nerve connections; and a very thorough bibliography and index. The text is well printed and readable, and there are a great number of figures and diagrams, 195 in 400 pages; many of these are photographs, and when one knows the difficulty of getting details to show in dissections, these photographs will be appreciated. Diagrams and lettering superimposed on actual photographs give a very useful and lasting effect. From a practical and operating point of view the lachrymal apparatus is perhaps the most important in the orbit, and the dissections and diagrams of this are thorough and original. A note is also made of the chief abnormalities met with. Just enough of the adjacent sinuses and other structures are described to throw light on the subjects which overlap to the Rhinologist and Neurologist. The book is well got up, thorough, and exhaustive, but not so exhausting as contributions of this nature are apt to be. A few radiographs from the living subject with the anatomical structures labelled might prove a useful addition to the next issue.

**Treatment of Injuries of the Peripheral Spinal Nerves.** By Sir Harold J. Stiles, K.B.E., F.R.C.S. Ed., and M. F. Forrester-Brown, M.S., M.D. Pp. viii., 180. London: Oxford Medical Publications. 1922. Price 15s.—This book is a very valuable contribution to our knowledge of injuries of nerves and their treatment, and is based on the very large experience of the authors at the Edinburgh War Hospital. Its special feature is the description of the method of operation for each nerve, with very clear and helpful illustrations. This very valuable portion of the book occupies almost two-thirds of it. The section which follows, on tendon and muscle transplantation in cases in which an operation on the nerve cannot be performed or fails, is also a most useful one, as the technique is fully given, and as in the previous section on operations on the nerves, it is well illustrated. The title of this book might lead us to expect that it only related to the treatment of nerve injuries, but the function of the various nerves and the diagnosis of their injuries are also considered, but perhaps not as fully as we should expect in a work dealing with every aspect of nerve injury. There are several points which have struck us as of interest in our perusal of this book. We note that the authors have not accepted Mr. Sherren's views as to the sensory supply of the musculo-spiral nerve, and we are not surprised at this, as we have found anæsthesia in the radial supply in injuries of the musculo-spiral nerve below the origin of its external cutaneous branches; and after removal of a portion of radial nerve in the upper third of its course, for grafting into a nerve gap, we produced a very definite anæsthesia in its supply. But we are rather surprised to see in Fig. 6*b* the representation of the median nerve cutaneous supply on the dorsum of the thumb. Sherren found there was no anæsthesia there in 41 cases of division of the median nerve which he had observed. And in Fig. 8*a* we are interested to see that the plantar nerves are represented as supplying an area on the dorsal aspect of the toes, just as the median does on the dorsal aspect of the fingers, but Sherren gives the nerve supply of the plantar nerves as only to the dorsal aspect of the outer four toes and not the great toe. The advice given with regard to operations under the heading "General Considerations" is very helpful. The authors favour earlier operation in cases of incomplete gunshot injury than many surgeons have advised. They do not advocate even waiting three months, in order to avoid the risk of reawakening sepsis in the wound, and say that if the application of firm, and then heavy, massage to the wound fails to light up any inflammatory reaction, operation may with advantage be undertaken within two or three weeks of the date of healing. In describing the methods which may be employed in dealing with gaps in the nerve, the authors agree with other recent writers on the late results of operations on nerves, that nerve grafting is very little good, and all they say in favour of it is that there is no harm in trying it if all other methods fail. Nerve transplantation they do not even mention, probably because, like nerve grafting, the late results have been so discouraging. But we must remember that in some cases fairly good results have been obtained in civil practice by nerve transplantation in the case of the divided facial nerve, and it seems to us that it might at any rate have been mentioned as a possible resort, for instance in dealing with injuries of the cords of the brachial plexus. The importance of a knowledge of where the branches of a nerve, the surgeon is about to attempt to unite, come off is emphasised, so that in freeing it, in order to lengthen it, for the purpose of bridging a gap, only the less important ones may be sacrificed. The two-stage operation for dealing with a wide gap in a nerve is fully described and illustrated, and is considered to be a valuable procedure. We notice that the order of recovery after reunion of a nerve differs from that given by Sherren. He taught in his book on *Injuries of Nerves* that there was first a return of protopathic sense only, and that this return was independent of the distance of the lesion from the periphery of the limb, and usually appeared about 6 to 16 weeks after union, but that the return of epicritic sense appeared later. There is no mention of this order of recovery in the work under review, and we gather from the description of the method of recovery that it has not been observed.

**The Oxford Index of Therapeutics.** Edited by VICTOR E. SORAPURE, M.B., Ch.B., F.R.C.S. Ed. Pp. xvi., 1126. London: Oxford Medical Publications. 1921. Price £2 2s. od.—This encyclopædic volume is intended for the use of the general practitioner both in America and Great Britain. The list of contributors of articles includes many of the best-known authorities on medicine and surgery in both countries. Thus in Great Britain we note the names of Poynton, Frederick Price, Sir Thomas Horder, Lockhart Mummery, Colonel Harrison, our late colleague Fortescue-Brickdale, William Turner, Agnes Savill and others. Some of the articles are treatises in themselves of great interest and value. The aim has been that each writer should supply an account of what in his opinion is the best method of treatment for the condition in question, rather than that he should describe a variety of suggested treatments. The success of this plan of course depends on the excellence of the staff whom the editor has collected. We were interested to see articles on proteid sensitization, chemotherapy, colloidal remedies, and specific bacterial therapy.

As a certain amount of overlapping is inevitable, the therapeutical index is a most important and essential part of the work, and must be constantly consulted.

**Manual of Physio-Therapeutics.** By THOMAS DAVEY LUKE, M.D., F.R.C.S. Ed. New and revised edition. Pp. xvi., 480. London: William Heinemann Ltd. 1922. Price 25s.—We warmly welcome the reappearance of this well-known treatise in its enlarged form. It meets a want, for we know of no other single volume which gives the essentials of all the various forms of physical treatment. One is surprised at the amount of information which the author has contrived to include in its pages. He is fortunate, too, in his illustrations, of which he has now over 200. It is doubtful whether it was worth while to attempt an account of Massage in the short space available, though it may be useful to have the primary rules for reference. The same remark applies even more strongly to the chapters on X-rays and Diathermy, which gives us little beyond a description of the apparatus used. On the other hand, the discussion of the technique of baths, and of physical exercises on the Bruce Sutherland system are excellent. We should like to see the need of the black bulb thermometer in light baths mentioned. There is much common sense in the chapters on Diet, but in the account of the Allen treatment of diabetes (page 465), after the first two days of fat restriction, a line descriptive of the absolute fast has apparently dropped out. The text goes on to say that " for 24 hours after the urine has become free of sugar," without directly showing how it has been made so, though various details of the process are given later on. In the reference to the effects of cooking on green vegetables (p. 419) the great waste in boiling them instead of steaming or stewing should be made clear. The death of the author has cut short at an early age a promising career.

**The Principles of Electrotherapy, and their Practical Application.** By W. J. TURRELL, M.A., D.M., B.Ch. (Oxon.). Pp. xi., 276. London: Oxford Medical Publications. 1922. Price 12s. 6d. net.—This is a work which should not be overlooked by anyone studying the development of medical electricity, for the writer's aim is to explain its therapeutic action on physical and physiological principles, and he reaches various conclusions of high importance. Besides this attempt to rationalise electrical theory he gives an account of much of the recent work and practice of French authorities which is little known in this country. His views on Ionic Medication are interesting. While he shows the impossibility of driving drugs deeply into the tissues so as to act chemically there, he argues that the actual success obtained by this method of treatment is due mainly to the heat generated in the tissues by the passage of the current and the chemical changes in the neighbourhood of the electrodes. He points out that the ordinary method of ionisation, though the theory is faulty, does enable us to apply a stronger current and for a longer time than any other plan, and this he thinks accounts for its acknowledged success. The brilliant results in the treatment of septic sinuses and endometritis are, he argues, really polar effects, and not due to deep ionisation. We should like to hear how his theory accounts for the actual removal of a weighable quantity of lead from the body by the constant current, as Sir Thomas Oliver has succeeded in doing. In the able account he gives of the action of Radiant Heat it is curious to find that he can discover practically no difference in the therapeutic effects of dark heat, light rays, incandescent lamps, hot sand and wax baths. Even in the sun baths the most powerful action is that of the infra-red rays. For a long time we have constantly had the superior effects of light rays over dark heat urged upon us, and Dain and others have given some experimental proofs of their greater power of raising the body temperature, but even now we can hardly regard the question as finally settled. One of the most interesting parts of the book is the discussion of the Erlangen treatment of cancer, and the stimulating, inhibitory, and destructive action on cells of various forms of radiations. He is quite clear as to the danger of prophylactic doses of X-rays before or after operation, since mild doses are likely to have a stimulative rather than a destructive effect. His rapid cure of sprains and acute synovitis by the Morton wave current and its use for massage of the prostate seems to have a practical importance, and we can confirm the value of his treatment of brachial neuritis by a strong galvanic or diathermic current over the cervical and upper dorsal vertebræ, where the seat of the trouble actually is.

**A Manual of Fevers.** By C. B. Ker, M.D. Pp. xiii., 334. London: Oxford Medical Publications. [1922.] Price 12s. 6d. net.—This is a second edition of an excellent work dealing with the infectious diseases usually admitted into fever hospitals. The main features of these diseases are briefly but lucidly dealt with. Every page shows evidence of the long personal experience of the author as a clinician and teacher in presenting his material in the manner most useful to the student, to whom one can cordially recommend the book. In discussing the differential diagnosis of diphtheria the author states that "quinsy should not give much trouble." This is a very misleading statement in regard to the types of the disease in certain epidemics. Two tables complete the manual.

**The Logic of the Unconscious Mind.** By M. K. BRADBY. Pp. xiv., 316. London: Oxford Medical Publications. 1920. Price 16s. net.—Miss Bradby's book is laborious reading and one is left wondering what it is all about. Meaning is largely obscured by looseness in terminology. There is, moreover, a general lack of coherence both in argument and arrangement. The first chapter, on Logic, could with advantage be condensed into a single page. The chapter on Instinct strikes one as chaotic. It contains no clear definition of instinct. Instinctive actions, the author tells us, are non-rational as regards intention, but rational or irrational as regards effect. She probably means that they appear rational or irrational to the onlooker. Miss Bradby seems to confuse instinct and sentiment We are told "one leading instinct in man is his love of truth for truth's sake. . . . This instinct impels to the pursuit of logic!" It is noteworthy that this chapter contains no reference to the works of McDougall or Shand. Intuition is treated as "another function of the mind." Surely it is simply an impulsive perception, which constitutes the cognitive side of the instinctive process. Again, the classification of fallacy is obscure and artificial. Does "fallacy necessarily arise from a lack of co-ordination amongst mental processes belonging to different levels of development?" Language, we are told, is the "magnum opus" of the unconscious mind. Surely it is the work of the conscious; words may have been originally unconscious symbols, but they are fitted together consciously to form language. It is difficult to see to what section of the reading public this book is likely to have any appeal.

**Golden Rules of Dental Mechanics.** By HAROLD OSBORN. Pp. 95. Bristol: John Wright & Sons, Ltd. [N.D.] Price 5s. net.—This little book contains many useful hints and much information which undoubtedly is valuable to junior mechanics, for whom it is presumably written. Many of the rules are by no means acceptable. For example, moist heat is advocated for packing, the oldest mechanics and practitioners prefer in most cases dry heat. The rules on casting are useful, but the author advocates the vacuum process, though this method is by no means accepted as the best. Some useful information is given at the end of the book on formulæ for solders, useful chemicals, etc. The book is well produced with a clear good type well arranged, and contains a useful index at the back.

